# Gut Bacteria Strategies of *Hylurgus ligniperda* F. (Coleoptera Scolytidae) in Adapting to Temperature Changes

**DOI:** 10.3390/microorganisms13112502

**Published:** 2025-10-31

**Authors:** Huanwen Chen, Dan Xie, Lihong Jiang, Fang Niu, Xiaomei Wang, Yan Dai, Defu Chi, Jia Yu

**Affiliations:** 1 College of Forestry, Northeast Forestry University, Harbin 150040, China; chenhuanwen2020@126.com (H.C.); xiedan0807@126.com (D.X.); jianglihong199@126.com (L.J.); niufang890526@126.com (F.N.); daiyan50062618@163.com (Y.D.); 2 National Permanent Scientiffc Research Base for Warm Temperate Zone Forestry of Jiulong Mountain in Beijing, Experimental Centre of Forestry in North China, Chinese Academy of Forestry, Beijing 100091, China; xiaomeiwang930425@outlook.com

**Keywords:** *Hylurgus ligniperda*, temperature, bacterial communities, culturable bacteria, nutrient conversion

## Abstract

Insect establishment and dispersal are often influenced by temperature, with gut microbiota playing a critical role in host adaptation to environmental stress. This study investigated how gut bacterial structure and function in the invasive red-haired bark beetle (RHB), *Hylurgus ligniperda* (Fabricius) respond to temperature fluctuations, focusing on three core culturable bacteria: *Rahnella perminowiae*, *Serratia marcescens*, and *Hafnia psychrotolerans*. We found that temperature variations induced specific structural changes in the gut bacterial community, which in turn affected key functional processes such as carbohydrate metabolism. Notably, the relative abundance of *Rahnella* increased by more than 10% during the cold period (CP), and it maintained stable production of proteases and lipases under low temperatures—a trait that may be crucial for supporting host development in cold environments. Feeding on the diet converted by *R. perminowiae* at 5 °C resulted in a 20.9-day reduction in pupation time and a 1.8-fold increase in adult body mass compared to the blank control group, respectively. We propose that temperature remodels the gut microbiota by modulating competitive relationships among functional bacteria. This regulatory mechanism, based on functional redundancy and dynamic balance, serves as a buffer strategy that aids insect adaptation to temperature changes. Our findings provide new insights and a theoretical foundation for understanding pest outbreak patterns under climate warming and developing microbe-targeted control strategies.

## 1. Introduction

Insects represent the most abundant, widely distributed, and diverse group of animals on Earth, with an evolutionary history spanning approximately 480 million years [1,2]. Throughout their long evolution, insects have developed remarkable diversity in development, behavior, social structure, and ecological functions, enabling them to adapt to a wide range of complex environments [3,4]. Currently, climate change is affecting global biodiversity and ecosystems on an unprecedented scale. As ectotherms, insects are highly sensitive to climatic shifts [5] and have evolved various strategies to cope with environmental change [6], including establishing symbiotic relationships with microorganisms [7,8]. These mutualistic mechanisms provide critical survival advantages under adverse conditions, and the functional redundancy of the gut microbiota further enhances their environmental adaptability.

The insect gut provides a unique habitat for microorganisms, with its community structure shaped by host species, habitat, and diet [9,10]. Even within the same insect species, the gut microbiota undergoes dynamic succession in response to dietary shifts and environmental changes [8,11]. The host provides a stable environment and nutrients for the gut microbes, which in turn participate in crucial physiological processes such as nutrient metabolism and detoxification [12,13]. These microbial functions significantly influence the host’s nutrition, development, and environmental adaptability [8,14]. The gut microbiota encompasses a wide range of functional groups. Functional redundancy within these communities enables the microbiota to maintain stability and assist the host in adapting to environmental fluctuations. Key functions of the gut microbiota include: supplying essential nutrients and amino acids (EAAs) for insect growth, digesting complex macromolecules, and degrading toxic plant secondary metabolites [15,16]. If specific functional groups are impaired by environmental stress, their associated physiological functions would be compromised, potentially leading to host developmental delays and reduced immunity. In summary, the insect gut microbiota constitutes a complex and multifunctional system that profoundly influences host phenotypes related to environmental adaptation, providing essential nutritional and protective benefits [17].

In recent years, pest management has grown increasingly complex due to the combined effects of climate change, rapid globalization, urbanization, and the spread of invasive species [18]. Forestry pests inflict substantial economic losses and pose severe ecological threats. As a representative group of forestry pests, bark beetles are distributed across global forest ecosystems and represent a major biotic driver of tree mortality in coniferous forests [19,20]. Outbreaks of bark beetles, often triggered by external environmental factors such as drought, climate warming, stand characteristics, and soil conditions, have caused widespread tree mortality, profoundly impacting ecosystem structure, function, and species composition [21]. Consequently, investigating how bark beetles adapt to environmental temperature—a critical abiotic factor—is crucial for understanding the mechanisms behind their outbreaks. This research also provides a scientific basis for predicting their potential distribution in invaded regions.

The red-haired pine bark beetle (RHB), *Hylurgus ligniperda* (Fabricius), is a globally significant invasive species native to Eurasia [22]. The RHB burrows into and feeds on the phloem of *Pinus* spp. [23], reducing the vitality of native pine forests, impairing their windbreak and sand-fixation capacity, and causing severe ecological damage. This pest has now successfully established populations on every continent where its host pines are distributed [24,25]. Research indicates that temperature is a key determinant of RHB’s geographical distribution, as its physiological processes and reproductive development are highly sensitive to thermal fluctuations [26,27]. Across transcontinental temperature gradients, the symbiotic system formed between the gut microbiota and RHB may be a critical factor facilitating the beetle’s global colonization. Although a previous study [28] investigated the microbial community structure across different anatomical parts of the RHB and performed functional predictions, the dynamic responses of the RHB gut microbiota to changing temperatures and the mechanisms by which they underpin the persistence of RHB populations in complex, fluctuating environments remain unclear. Elucidating this aspect will not only enhance our understanding of how symbiotic microbes maintain insect health but could also advance management strategies for invasive species.

This study investigates the role of gut bacterial community in facilitating RHB’s adaptation to environmental temperature shifts. Using established RHB populations in China, we applied metagenomic approaches to analyze the composition and functional dynamics of its gut bacterial communities across seasons with drastic temperature fluctuations. Furthermore, we experimentally validated the functions of temperature-sensitive bacterial isolates and their impact on RHB growth and development. Our findings establish a theoretical framework for understanding the synergistic mechanisms between gut bacterial symbionts and RHB development under significant temperature variation and provide a scientific basis for developing more effective pest control strategies.

## 2. Materials and Methods

### 2.1. Insect Collection

Adult *Hylurgus ligniperda* (RHB) beetles were collected from coastal shelterbelt forests in Muping District, Yantai City, Shandong Province, China (37°44′ to 37°45′ N, 121°72′ to 121°85′ E). Sampling was conducted during early September 2023 (warm period, WP), late November 2023 (cooling period, CIP), mid-January 2024 (cold period, CP), and late March 2024 (warming period, WIP). During each sampling period, five recently infested stumps of thinned *Pinus thunbergii* with fresh frass were selected in the forest. The temperature of the phloem and cambial region in each stump was measured using a handheld temperature recorder (Shandong Renke Control Technology Co., Ltd., Jinan, China). After carefully removing the bark, adult RHBs were extracted from their galleries using forceps and placed into sterile 50 mL centrifuge tubes. A total of 150 healthy and active adults were collected from the five stumps per sampling event. The tubes containing the beetles were then returned to the exposed phloem-cambial interface and covered with the original bark. Following a 24 h starvation period under field conditions, the specimens were flash-frozen in liquid nitrogen, transported on dry ice, and subsequently stored at −80 °C until subsequent analysis.

### 2.2. Gut Sample Preparation and DNA Extraction

Adult RHB specimens collected from the phloem and cambial regions of field stumps were subjected to dissection. Adult beetles were selected for this procedure because this developmental stage predominates within these tree regions during field collection. The dissection protocol involved the following sequential sterilization steps: first, rinsing the adult beetles’ surfaces with sterile water; then immersing them in 0.5% sodium hypochlorite solution for 5 min; followed by disinfection with 75% ethanol for 2 min; and ultimately, a final rinse with sterile water for 1 min. The sterilized adults were blotted dry on sterilized filter paper before being transferred to a laminar flow hood (Harbin Donglian Electronic Technology Development Co., Ltd., Harbin, China) for dissection. The complete intestinal tract was carefully excised from each specimen and placed in sterile 2 mL centrifuge tubes for subsequent total DNA extraction. The primary objective of this research was to investigate the influence of gut bacteria on the development of RHBs. Therefore, during the preparation of gut samples, males and females were not separated; instead, they were processed as pooled samples with a controlled sex ratio of 1:1. Total genomic DNA of gut microbiota was extracted using the TGuide S96 Magnetic Bead-Based Genomic DNA Extraction Kit (TIANGEN, Beijing, China). The concentration and purity of amplicons were measured using Qubit 3.0 (Invitrogen, Shanghai, China), Nanodrop 2000 (Thermo Fisher Scientific, Shanghai, China), and agarose gel electrophoresis, respectively. The qualified DNA was sent to Biomarker Technologies for amplicon sequencing using the Hiseq 2500 PE250 metagenome sequencing system.

### 2.3. Metagenome Sequencing and Annotation

Prior to DNA sequencing, a library was constructed using NEBNext^®^ UltraTM DNA Library Prep Kit for Illumina (NEB, Ipswich, MA, USA) following the manufacturer’s instructions. The raw reads containing three or more “N” or contaminated by adapters (>15 bp overlap) were removed, and the filtered clean reads (about 6.0–8.5 Gb per sample) were used for metagenomic analyses. The metagenomic assembly was performed using Megahit [29] in default mode. MetaGeneMark (v 2.10) was employed to predict genes from the assembled contigs, and redundancy was removed using CD-HIT Software version 4.8.1 [30].

On the platform BMKCloud (www.biocloud.net, accessed on 15 February 2025), the data were examined. The functional annotations were conducted by aligning them to the following databases using DIAMOND v0.8.35 (http://www.diamondsearch.org/ index.php, version 0.8.35, accessed on 18 March 2025) [31]: NCBI non-redundant protein sequences (nr) (for taxonomy annotation of metagenomic contigs and functional annotation for the CDS of bins); Kyoto Encyclopedia of Genes and Genomes (KEGG), with an e-value cut-off of 1 × 10^−5^. In taxonomic analysis, sequences annotated as those from Metazoa and Viridiplantae were excluded. In functional analysis, only sequences from microorganisms were included.

### 2.4. Screening of Culturable Gut Bacterial Strains

Culturable bacterial strains were previously isolated from the RHB gut (unpublished data) by pooling and homogenizing the guts of 30 adult beetles, followed by serial dilution and spread-plating for isolation and identification. Following this, a comparison with gut microbial communities from different temperature regimes identified bacterial genera with significant differential abundance. The present study focused on three selected strains from this effort: Rahnella perminowiae (accession no. PP783847.1), Serratia marcescens (accession no. PQ670071), and Hafnia psychrotolerans (accession no. PQ670078). These strains are maintained in the Key Laboratory for Sustainable Forest Ecosystem Management of the Ministry of Education, College of Forestry, Northeast Forestry University. The selected bacterial strains, stored at ultra-low temperatures, were first streaked onto Luria–Bertani (LB) agar plates and incubated at 25 °C for 36 h. Subsequently, bacterial disks (3 mm in diameter) were aseptically harvested from these cultures using a sterile cork borer. Each disk was inoculated onto a separate plate of screening medium containing a specific substrate, with one disk per plate. All bacterial strains were incubated at three different temperatures: 5 °C, 15 °C, and 25 °C. For each strain–temperature combination, three replicate plates were prepared. Lipase and protease activities were assessed after 48 h of incubation on their respective specific media. Enzymatic activity (E) was quantified and compared using the formula E = D/d, where D is the diameter of the hydrolysis zone (halo), and d is the diameter of the bacterial colony.

### 2.5. Impact of Gut Bacteria on Feed Nutrient Composition and RHB Development

Bacterial strains exhibiting both lipase and protease activities were inoculated into LB liquid medium. The cultures were incubated at 30 °C with shaking at 180 rpm for 24 h. Subsequently, 1 mL of the bacterial culture was transferred to a 1.5 mL sterile microcentrifuge tube and centrifuged at 5000× *g* for 2 min. The pellet was collected and resuspended in 1 mL of sterile distilled water to prepare a stock suspension. The stock suspension was then adjusted with sterile water to an optical density of 1.0 at 600 nm (OD_600_). The artificial diet was dispensed into 90 mm Petri dishes (30 g per dish) and sterilized by autoclaving at 121 °C for 20 min. Under aseptic conditions, 1 mL of the standardized bacterial suspension was spread evenly onto the surface of the sterilized artificial diet using a sterile glass spreader. The inoculated plates were incubated at a constant 5 °C, 15 °C and 25 °C for 7 days, followed by sterilization in an autoclave (121 °C, 20 min). Each treatment was prepared in triplicate. The control group received 1 mL of sterile distilled water. After incubation, 0.1 g samples were aseptically collected from each plate into 1.5 mL microcentrifuge tubes for subsequent determination of free amino acid and fatty acid content. The remaining sterilized artificial diet, which had been inoculated with gut bacteria and incubated for 7 days, was sealed and stored at 4 °C for subsequent insect rearing experiments.

Adult RHB individuals, captured using plant-derived attractants and traps, were transferred into black rearing containers (17 cm × 10 cm) containing fresh *Pinus thunbergii* phloem tissue. The containers were maintained in a climate-controlled chamber (Harbin Donglian Electronic Technology Development Co., Ltd., Harbin, China) set at 25 ± 1 °C, 60% relative humidity, and a 12 h light/12 h dark photoperiod to facilitate oviposition. The phloem tissue was replaced every 5 days. Collected RHB eggs were surface-sterilized by sequential immersion in the following solutions: 0.1% sodium hypochlorite for 5 min, sterile water for 2 min, 75% ethanol for 4 min, and a final rinse in sterile water for 2 min, followed by drying on sterile filter paper. Under aseptic conditions, the sterilized eggs (20 eggs per plate) were transferred onto the surface of the sterilized artificial diet that had been previously inoculated with gut bacteria and incubated for 7 days (as described in the preceding paragraph). Throughout the entire artificial rearing process of the RHB, the Petri dishes remained unopened to maintain a sterile environment for the diet. Each Petri dish was sealed with a 5 cm wide strip of Parafilm and maintained under the aforementioned environmental conditions. The date of pupation was recorded daily. Upon eclosion, adults were immediately collected, their body mass was recorded, and the content of soluble proteins and free fatty acids in the emerged adults was quantified.

The nutritional conversion of the formulated diet by gut bacteria was assessed by quantifying the levels of free amino acids and free fatty acids. Furthermore, the energy reserves of adult RHBs were evaluated by measuring their soluble protein and free fatty acid content. The contents of free amino acids (G0415W), soluble proteins (G0418W), and free fatty acids (G0927W96) were determined according to the manufacturer’s instructions (Grace Biotechnology Co., Ltd., Suzhou, China). An Epoch-cn microplate reader (Agilent ioTek, Winooski, VT, USA) was used to determine the absorbance of the reaction fluid of the nutrients. Each assay was performed in triplicate and repeated three times. The results were calculated according to the manufacturer’s instructions.

### 2.6. Culture Media Formulations and Artificial Diet Formulations for RHBs

The composition of each culture medium used in this study is as follows:

Luria–Bertani (LB) Agar: Prepared with 10.0 g/L peptone, 3.0 g/L beef extract, 5.0 g/L NaCl, and 20.0 g/L agar, adjusted to a final volume of 1000 mL with distilled water.

LB Broth: Prepared with 5.0 g/L peptone, 1.5 g/L beef extract, and 2.5 g/L NaCl, adjusted to a final volume of 500 mL with distilled water.

Lipase Screening Medium: Prepared with 10.0 g/L peptone, 5.0 g/L NaCl, 10.0 g/L Tween-20, and 15.0 g/L agar, supplemented with 0.1 g/L CaCl_2_·2H_2_O and 1.0 mL/L of a 1.6% neutral red solution.

Protease Screening Medium: Formulated with 10.0 g/L casein, 5.0 g/L peptone, 2.5 g/L yeast extract, and 20.0 g/L agar, along with 0.4 g/L KH_2_PO_4_, 1.0 g/L NaCl, and 0.5 g/L MgSO_4_.

The artificial diet was formulated as follows: 100 g phloem tissue, 0.5 g brewer’s yeast, 5 g agar, and 500 mL distilled water.

### 2.7. Statistical Analysis

The data were analyzed using SPSS 19 (IBM Corporation, Armonk, NY, USA), and the results are presented as means ± standard deviations. Statistical significance was assessed using one-way analysis of variance (ANOVA) and two-way ANOVA followed by Tukey’s honest significant difference test for multiple comparisons. Graphs were created using GraphPad Prism 8.0 (GraphPad Software, La Jolla, CA, USA). The correlation between stump temperature and the microbial community structure was analyzed using Pearson’s correlation coefficient.

## 3. Results

### 3.1. Gut Bacterial Community States in RHBs Under Different Temperature Conditions

**Figure 1:**
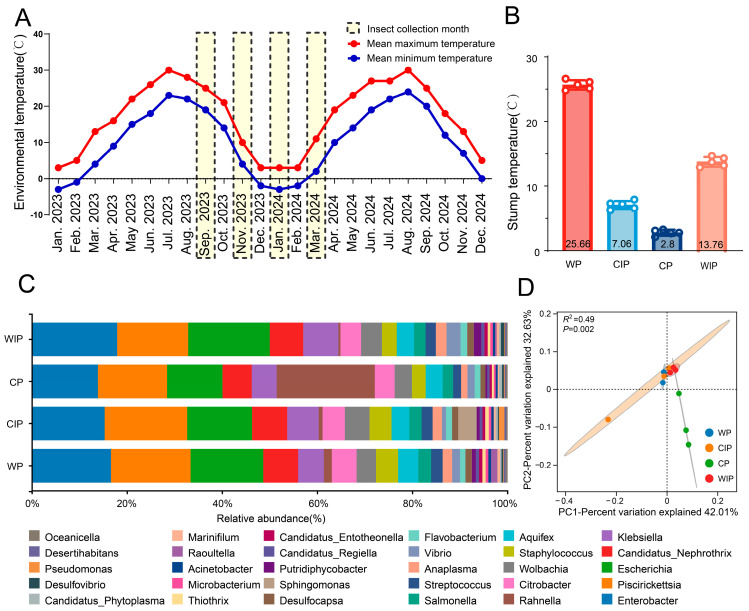
Gut bacterial community composition in RHBs under different temperature regimes. (**A**) Annual temperature variation profile at the RHB sample collection site. Meteorological data were sourced from the China Meteorological Administration (https://www.cma.gov.cn/, accessed on 13 January 2024). (**B**) Stump temperature recorded during RHB sampling. The open circles indicate the distribution of the data. (**C**) Bar plot showing the relative abundance of the top 30 dominant bacterial genera in the RHB gut across different temperature periods. (**D**) Dissimilarity in gut bacterial community composition under different temperature states, visualized by principal coordinate analysis (PCoA) based on the Bray–Curtis distance metric. The dashed lines indicate the origin (0,0). Circles represent individual samples, with closer circles indicating more similar community compositions. Shaded ellipses represent the 95% confidence ellipse for each group. Statistical significance of differences in microbial communities across temperature states was computed using PERMANOVA (via the adonis test). WP: Warm period; CIP: Cooling period; CP: Cold period; WIP: Warming period.

To decipher the dynamic changes in host gut bacterial composition in response to temperature variation, this study first showed the annual temperature profile in the RHB colonization zone. Four representative months were selected, and the gut bacterial communities of RHBs collected during these months were analyzed (Figure 1). Results demonstrated that the minimum survival temperature for RHBs throughout its developmental cycle was 2.8 ± 0.5 °C, with overall stump temperatures being slightly higher than ambient temperatures (Figure 1A,B). The taxonomic composition of gut bacteria at the genus level varied across different temperature phases (Figure 1C). Principal coordinate analysis revealed that bacterial communities formed distinct clusters under each temperature condition (PERMANOVA test, adonis, R^2^ = 0.49, *p* = 0.002). Overall, bacterial communities from the warm period (WP) and cooling period (CIP) showed a close association, while those from the CIP, cold period (CP), and warming period (WIP) also exhibited similarity. In contrast, the WP community was clearly separated from those of the CIP, CP, and WIP. Analysis of gut bacterial genus abundance under different temperature states (Figure 1C; Table S1) identified *Piscirickettsia*, *Enterobacter*, and *Escherichia* as highly abundant across all sampling time points, constituting the core taxa. Specifically, the top three genera with the highest relative abundance in the WP were *Piscirickettsia* (15.12%), *Enterobacter* (14.97%), and *Escherichia* (13.82%); in the CIP, they were *Piscirickettsia* (14.57%), *Enterobacter* (12.81%), and *Escherichia* (11.46%); in the CP, they were *Rahnella* (18.96%), *Piscirickettsia* (13.36%), and *Enterobacter* (12.69%); and in the WIP, they were *Enterobacter* (16.02%), *Escherichia* (15.36%), and *Piscirickettsia* (13.38%).

### 3.2. Metabolic Function and Key Genera of the RHB Gut Bacterial Under Different Temperature Regimes

**Figure 2:**
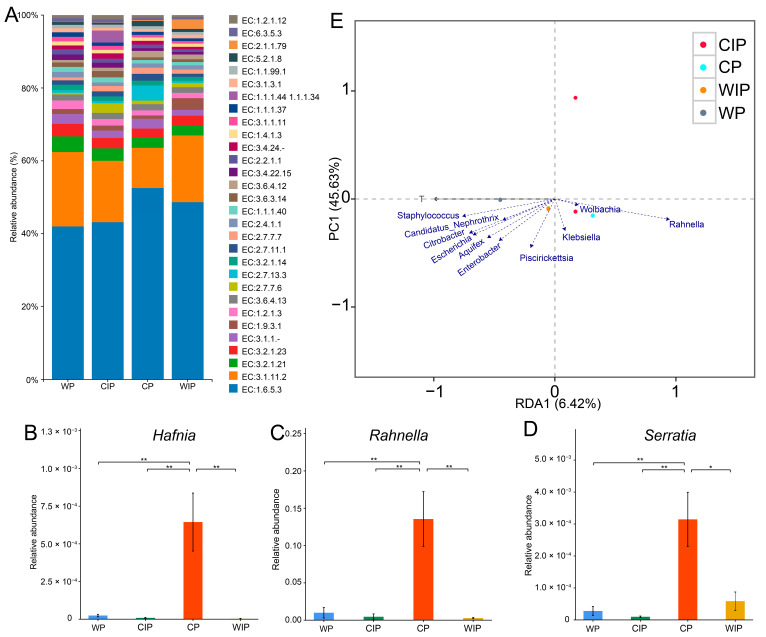
KEGG Enzyme category profiling and differential bacterial genera in the gut of RHBs under different temperature regimes. (**A**) Bar chart displaying the top 30 KEGG metabolic categories of gut bacteria in RHBs across temperature periods. The category “E: 3” is highlighted with a red underline. Relative abundance of (**B**) *Hafnia*, (**C**) *Rahnella*, and (**D**) *Serratia* under different temperature conditions, analyzed using one-way ANOVA (* *p* < 0.05, ** *p* < 0.01). (**E**) Correlation analysis between stump temperature and gut bacterial communities.

Analysis based on KEGG Enzyme classification indicated that hydrolases represented one of the core functional categories of gut bacteria in RHBs across different temperature regimes, accounting for approximately 40% of the top 30 Enzyme categories (Figure 2A). This finding suggests that gut bacteria in RHBs primarily employ hydrolytic enzymes to respond to ambient temperature changes. In our prior work, 23 culturable bacterial genera were isolated from the RHB gut. In the current study, these cultivable genera were cross-referenced with the differentially abundant bacterial genera identified in the RHB gut under various temperature states (Table S2). The results demonstrated significant variations in the relative abundances of *Hafnia*, *Rahnella*, and *Serratia* across the different temperature regimes. Specifically, the relative abundances of *Hafnia*, *Rahnella*, and *Serratia* were highest during the cold period (CP) and were significantly different from those in all other temperature phases (Figure 2B–D; one-way ANOVA, *p* < 0.05). Among these genera, *Rahnella* exhibited the most substantial increase, with its relative abundance rising by over 10% under CP conditions compared to other temperatures. We further utilized Redundancy Analysis (RDA) to explore the association between stump temperature and the gut bacterial community (Figure 2E). The RDA indicated a considerable influence of stump temperature on *Rahnella*, revealing a negative correlation between stump temperature and its relative abundance. Together, these results demonstrate that temperature variation influences the composition of the host’s gut bacterial community.

### 3.3. Screening for Culturable Gut Bacteria with Extracellular Protease and Lipase Activity

**Figure 3:**
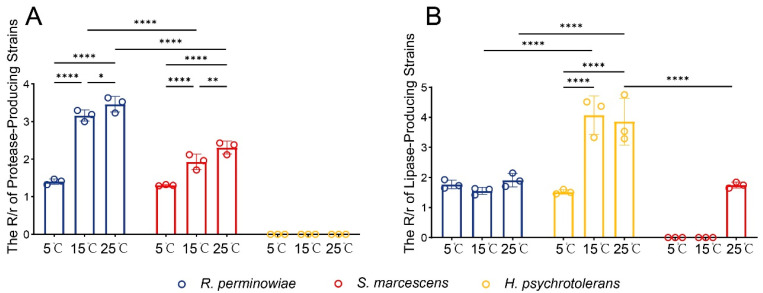
Screening of culturable gut bacteria for protease. The control group, which showed no hydrolysis zone (R = 0), was not indicated in the bar graph. (**A**) and lipase (**B**) secretion. Data were analyzed using two-way ANOVA (* *p* < 0.05, ** *p* < 0.01, **** *p* < 0.0001). Bacterial strains lacking enzyme production capability were excluded from the differential analysis. The black lines represent significant relationships between the connected bars, and the open circles indicate the distribution of the data.

Given the significant differences in the relative abundance of *Hafnia*, *Rahnella*, and *Serratia* across different temperature regimes, strains of *H. psychrotolerans*, *R. perminowiae*, and *S. marcescens* isolated from adult RHBs were selected for screening of extracellular protease and lipase production. The results for extracellular protease secretion are shown in Figure 3A. *H. psychrotolerans* did not exhibit protease secretion capability at 5 °C, 15 °C, or 25 °C. In contrast, both *R. perminowiae* and *S. marcescens* demonstrated the ability to secrete proteases at all three temperatures. The R/r ratios for both *R. perminowiae* and *S. marcescens* were highest at 25 °C, significantly exceeding those at 5 °C and 15 °C (two-way ANOVA, *p* < 0.05), and decreased with declining temperature. Furthermore, at both 15 °C and 25 °C, the R/r ratio of *R. perminowiae* was significantly higher than that of *S. marcescens* (two-way ANOVA, *p* < 0.05). Regarding extracellular lipase secretion (Figure 3B), *S. marcescens* secreted lipases only at 25 °C. Both *R. perminowiae* and *H. psychrotolerans* secreted lipases at 5 °C, 15 °C, and 25 °C. The R/r ratio for *R. perminowiae* did not differ significantly across the three temperatures, with values at 5 °C being higher than at 15 °C but lower than at 25 °C. For *H. psychrotolerans*, the R/r ratio was highest at 15 °C and lowest at 5 °C, being significantly lower than the ratios at 15 °C and 25 °C (two-way ANOVA, *p* < 0.05). At 25 °C, the R/r ratio of *H. psychrotolerans* was significantly higher than those of both *S. marcescens* and *R. perminowiae*. At 15 °C, the R/r ratio of *H. psychrotolerans* was significantly higher than that of *R. perminowiae* (two-way ANOVA, *p* < 0.05). These results indicate that the capacity of the bacterial strains to secrete extracellular proteases and lipases is markedly influenced by temperature.

### 3.4. R. perminowiae Impact on Feed Nutritional Structure and RHB Development at Three Different Temperatures

**Figure 4:**
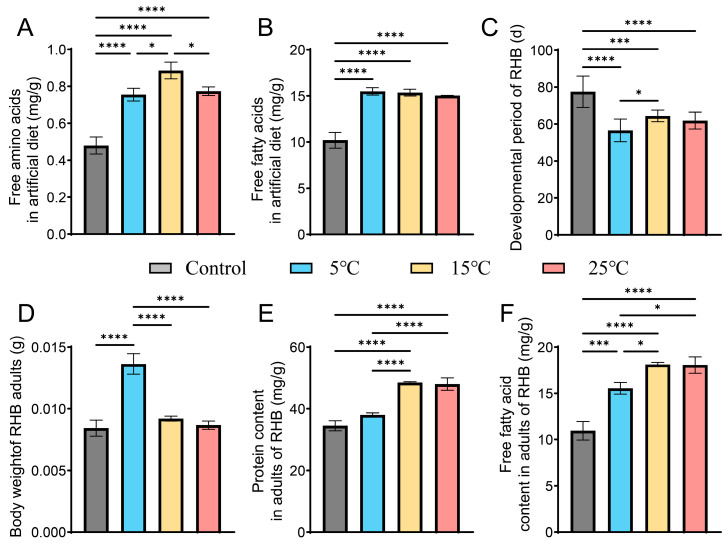
Effects of *R*. *perminowiae* on feed nutrient composition and RHB development three different temperatures. (**A**) Free amino acid content in the artificial diet following inoculation with *R. perminowiae*. (**B**) Free fatty acid content in the artificial diet following inoculation with *R. perminowiae*. (**C**) Duration of development from egg to pupa in RHBs fed the *R. perminowiae*-inoculated diet. (**D**) Body mass of adult RHBs fed the *R. perminowiae*-inoculated diet. (**E**) Soluble protein content in adult RHBs fed the *R. perminowiae*-inoculated diet. (**F**) Free fatty acid content in adult RHBs fed the *R. perminowiae*-inoculated diet. Data were analyzed using one-way ANOVA (* *p* < 0.05, *** *p* < 0.001, **** *p* < 0.0001).

*Rahnella perminowiae* was selected for subsequent experiments to investigate its impact on the nutrient composition of the artificial diet after incubation at three different temperatures, as well as its effects on host growth and development (Figure 4). This selection was based on its dual capability to secrete both extracellular proteases and lipases, coupled with correlation analyses indicating that stump temperature significantly influenced the abundance of *Rahnella* within the gut bacterial community.

Our results demonstrate that the diet treated with *R. perminowiae* facilitated RHB development by altering its nutritional structure. Specifically, RHB feeding on the diet incubated at 5 °C experienced a mean reduction in developmental duration of 20.9 days, which was significantly greater than that observed in the blank control group and the group fed with diet incubated at 15 °C (Figure 4A–C). Furthermore, the diet metabolized at this temperature enabled emerging adults to achieve a significantly higher body mass, approximately 1.6 times that of the control group, and significantly greater than other treatment groups (Figure 4D).

The diet incubated at 15 °C contained a significantly higher free amino acid content—about 1.8 times that of the blank control—which was also significantly different from other groups (Figure 4A). The effects of the diet incubated at 25 °C on both the artificial diet itself and on RHB development were highly similar to those of the diet incubated at 15 °C, showing no significant differences in most metrics except for free amino acids. Although no significant differences in free fatty acid content were detected among the temperature-treated diets, all treatment groups exhibited levels significantly higher than the control group. Diets transformed by *R. perminowiae* provided emerging adults with a higher proportion of protein and free fatty acids (Figure 4E,F).

These findings indicate that *Rahnella perminowiae* plays a crucial role in decomposing proteins and fats. The breakdown of these macromolecular nutrients is essential for the growth and development of the host.

## 4. Discussion

Insect sensitivity to environmental changes significantly influences the structural characteristics of their resident microbial communities. These communities serve as a critical bridge connecting the external environment with the host’s internal physiological state [32,33]. Leveraging natural environmental fluctuations and building upon the documented role of RHB gut bacteria in carbohydrate decomposition, this study investigated the mechanism through which ambient temperature, as a key abiotic factor, facilitates RHB adaptation by regulating its gut microbiota. The investigation focused on three culturable bacterial strains isolated from the red-haired bark beetle (RHB) gut: *Rahnella perminowiae*, *Serratia marcescens*, and *Hafnia psychrotolerans*. Our findings reveal that discernible variations occur in the gut microbial composition of RHBs across different phases of temperature variation within its habitat, with the relative abundance of *Rahnella* exhibiting a notable fluctuation of approximately 10–15%. These compositional shifts may potentially compensate for temperature-induced functional impairments or lead to alterations in the functional outputs mediated by specific microbial consortia, thereby facilitating adaptation to fluctuating habitat temperatures.

Climate is a key environmental factor shaping the global structure of insect gut bacterial communities [34]. The cross-border dispersal of invasive species often triggers regional ecological crises, with their successful colonization hinging on the insect’s adaptability to both environmental conditions and host plants. *Pinus* tissues are relatively nutrient-poor, and bark beetles exhibit low efficiency in absorbing nutrients from phloem. In this context, their symbiotic microbiota play a crucial role in facilitating digestion and nutrient absorption [35,36]. The assembly of gut microbial communities, filtered by microenvironmental factors, represents a common ecological phenomenon in insects. For instance, interspecies bacterial competition regulates community assembly in the *Caenorhabditis elegans* intestine [37]; pesticide exposure likewise impacts microbiota assembly in insects [38]; and Benjamin et al. demonstrated that temperature regimes distinctly influence microbial community characteristics using *Escherichia coli* [39]. These microbial communities generally exhibit remarkable environmental adaptability, capable of restructuring themselves in response to habitat changes [40]. In constructing synthetic microbial communities (SynComs), temperature is frequently employed as a key regulatory factor [41]. Among the three RHB gut bacteria investigated, *Rahnella perminowiae* demonstrated remarkable thermal adaptability. While its enzyme production at 15–25 °C was lower than that of *S. marcescens* and *H. psychrotolerans*, it maintained stable enzymatic output under low-temperature conditions, thereby supporting RHB development during cold periods. The robust enzyme production exhibited by *Rahnella perminowiae* under low-temperature conditions may be attributed to its exceptional cold shock proteins [42]. The observed increase in its relative abundance likely stems from this genus’s superior temperature adaptability. Under low-temperature conditions, its key physiological functions—such as nutrient utilization efficiency—were markedly superior to those of other taxa within the community, thereby securing a competitive ecological advantage and establishing it as a dominant, functionally significant population. Given the substantial climatic variations across continents, the symbiotic relationship forged between insects and their gut bacteria may well have facilitated the global successful colonization of RHBs to some extent.

Many insects can adjust their physiological functions in response to varying temperatures, thereby enhancing their survival capacity [43,44]. Advances in metagenomic sequencing have progressively revealed the diversity and functional roles of insect gut microbiota. When the temperature of an insect’s microenvironment changes, the intestinal temperature shifts accordingly, directly triggering dynamic restructuring of the microbial community. This may allow temperature-adapted strains to rapidly increase their biomass, while non-adapted taxa are temporarily suppressed until environmental conditions stabilize. This symbiotic mechanism—underpinned by functional redundancy and integration with environmental microbial resources—significantly strengthens the insect’s adaptability to thermal fluctuations. However, overgrowth of certain symbiotic microbial populations may also lead to their transition into pathogens [45,46]. During temperature-driven microbial restructuring, different bacterial species compete and replace one another according to their optimal growth temperatures and substrate preferences. Such fine-scale adjustments in the gut microbial community enable functional adaptation to environmental changes without causing substantial functional imbalance [47]. The periodic fluctuation of diurnal temperature variations in natural habitats maintains this microbial regulation in a dynamic equilibrium, guiding insect adaptation while simultaneously preventing severe disruption of the microbial community.

This study reveals that the gut microbiota serves as a key facilitator enabling bark beetles to respond to temperature variations and achieve colonization. This finding transcends the traditional paradigm of explaining environmental adaptability solely through host genetics, and establishes a new model wherein the host–microbiota holobiont functions as an integrated unit to cope with thermal changes. The proposed “temperature–microbiota–host” regulatory framework exhibits strong universality, providing not only a novel paradigm for interpreting outbreak dynamics of wood-boring pests under global warming, but also a unified perspective for understanding insect responses to multifaceted stressors such as drought and pesticide exposure. The underlying mechanisms provide a theoretical foundation for developing microbe-targeted ecological regulation strategies, offering critical insights for optimizing pest management and advancing biological control systems.

In summary, this study, by deciphering the dynamic functional characteristics of the gut microbiota in the RHB in response to temperature fluctuations and linking these dynamics to the synergistic contribution of bacteria to RHB development, has elucidated the potential role of gut microbes in collaboratively assisting the host to adapt to thermal changes. It provides mechanistic insights at a microscopic level for predicting insect population dynamics under climate change. However, the functional roles of gut microbial communities in supporting host survival are not singular; their inherent functional complexity and the coordination among different functional consortia warrant further investigation. Furthermore, the resilience of these gut functional communities across distinct geographical populations requires additional study. The value of this work lies in establishing a theoretical foundation and an analytical framework. Subsequent research can build upon this theoretical basis to develop microbiome-based strategies for ecological regulation, aiming to enhance the climate resilience of beneficial insects or constrain the distribution range of pest species.

## Data Availability

The original contributions presented in this study are included in the article/Supplementary Material. The gut bacteria metagenomic data have been deposited in the National Microbiology Data Center (NMDC) with the accession numbers NMDC40092158 (https://nmdc.cn/resource/genomics/metagenome/detail/NMDC40092158). Further inquiries can be directed to the corresponding author(s).

## Supplementary Materials

The following supporting information can be downloaded at: https://www.mdpi.com/article/10.3390/microorganisms13112502/s1, Table S1: Taxonomy.genus.relabunda; Table S2: Differential bacterial genera in the gut.
